# Research Progress towards the Effects of Fatty Acids on the Differentiation and Maturation of Human Induced Pluripotent Stem Cells into Cardiomyocytes

**DOI:** 10.31083/j.rcm2403069

**Published:** 2023-02-24

**Authors:** Zhen GAO, Fan ZHOU, Junsheng MU

**Affiliations:** ^1^Department of Cardiac Surgery, Capital Medical University Affiliated Beijing Anzhen Hospital, Beijing Institute of Heart Lung and Blood Vessel Diseases, 100029 Beijing, China; ^2^Department of Ultrasound, The Third Medical Center of PLA General Hospital, 100039 Beijing, China

**Keywords:** fatty acids, induced pluripotent stem cells, myocardial cells, cardiovascular disease

## Abstract

The incidence of cardiovascular disease has been continuously increasing. Because cardiomyocytes (CM) are non-renewable cells, it is difficult to find appropriate CM sources to repair injured hearts. Research of human induced pluripotent stem cell (hiPSC) differentiation and maturation into CM has been invaluable for the treatment of heart diseases. The use of hiPSCs as regenerative therapy allows for the treatment of many diseases that cannot be cured, including progressive heart failure. This review contributes to the study of cardiac repair and targeted treatment of cardiovascular diseases at the cytological level. Recent studies have shown that for differentiation and maturation of hiPSCs into CMs, fatty acids have a strong influence on cellular metabolism, organelle development, expression of specific genes, and functional performance. This review describes the recent research progress on how fatty acids affect the differentiation of hiPSCs into CMs and their maturation.

## 1. Introduction

Human induced pluripotent stem cell (hiPSC) can self-renew and, under specific conditions, can differentiate into various kinds of cells. These cells are a current focus of stem cell research. All cell types in the body can differentiate into hiPSCs, which in turn form all tissues and organs. Therefore, the study of pluripotent stem cells has great potential for applications in organ regeneration and repair as well as disease treatment. Culturing hiPSC under certain conditions, such as CM maturation medium with fatty acids, applying different electrical stimulation [1, 2], and applying mechanical stretch [3], can improve hiPSC-CM maturation in some fields, such as developing sarcomere organization, improving contractility of hiPSC-CMs, and enhancing CM maturation-related gene expression [4]. In addition, as a result of metabolic maturation in low glucose solutions and high oxidative substrate media; hiPSC-CMs becomes susceptible to cellular damage, which is crucial to developing valid *in vitro* cardiac ischemia models [5]. CMs are the basic cells that form heart tissue [6]. They form cardiac fibers and are a part of the striated muscle with the ability to excite and contract. Fatty acids include three elements: oxygen, hydrogen, and carbon, which are the main ingredients of neutral fats, phospholipids, and glycolipids. Fatty acids’ role in the development of hiPSCs into CMs as a significant source of energy metabolism or as an independent exogenous source is irreplaceable. Different types of fatty acids, differences in the contents of fatty acids, as well as differences in the metabolism of fatty acids affect the differentiation and maturation of hiPSCs into hiPSC-CMs via different mechanisms [7, 8, 9]. By compiling, analyzing, and reviewing the recent literature, we present the influence of various fatty acids on the differentiation of human stem cells.

## 2. The Effect of Fatty Acids on Stem Cell Differentiation into Mature CMs

HiPSC-CMs are a useful source of cells to model diseases and regenerate myocardium. However, they exhibit fetal CM-like characteristics in terms of both cellular and metabolic functions and differ from adult CMs. During CM maturation, the function of mitochondrial oxidative enzymes is enhanced, and the source of energy for CMs converts progressively from glycolysis to β-oxidation of fatty acids. During the differentiation of hiPSC into CMs, the purity and maturation of hiPSC-CMs using fatty acids as the primary metabolic substrate has been demonstrated as follows. First, the presence of CM-specific markers, such as troponin T and sodium-potassium channels, indicates that the cells exhibit mature adult CM-like characteristics, as demonstrated by real-time quantitative polymerase chain reaction (RT-qPCR), immunoblotting, immunofluorescence, and electron microscopy. Second, cellular energy metabolism profiles are obtained by the XF96 Cell Extrapolation Analyzer, which determines the rate of oxygen consumption (ORC, pmol/min/ug protein) and extracellular acidification (ECAR, mpH/min/g of protein) to evaluate mitochondrial oxidation and glycolysis. These methods have demonstrated that CMs derived from hiPSC, in which fatty acids were the primary metabolic substrate, exhibit increased elongation, an increased number of mitochondria, more neatly aligned Z-lines, and developed expression of adult-like CM-associated genes [10]. These data suggest that a medium containing fatty acids enhances hiPSC-CM maturation. In addition, oxygen consumption rates associated with basal respiration, production of ATP, maximal respiration, and reserve respiratory ability (representing mitochondrial function), improve in hiPSC-CM using fatty acids as the primary metabolic substrate. Mature hiPSC-CMs exhibit greater changes in basal and maximal respiration because of the use of extrinsic fatty acids (palmin) in comparison with immature controls [1]. Fatty acid treatment improves metabolic maturation of hiPSC-CMs, primarily by increasing their number and mitochondrial oxidative function [1, 11].

The hypoxia-inducible factor (HIF)-la-lactate dehydrogenase Axon Axon A axis alterations in hiPSC-CMs prevents metabolic maturation. However, the addition of fatty acids shifts the primary metabolic mode of hiPSC-CMs by reducing aerobic glycolysis to promote oxidative phosphorylation and inhibit hypoxia inducible factor-1α (HIF-1α). Hypoxia inducible factor-1β (HIF-1β) inhibition promotes oxidative phosphorylation while inhibiting aerobic glycolysis, resulting in an increase in the number of mitochondria as well as cellular ATP content, which improves CM gene expression as well as sarcomeric length and contractility [12]. Conversely, unlike adult CMs *in vivo*, hiPSC-CMs in standard culture medium maintain an immature phenotype [13]. Supplementation of palmitate or oleate, which are fatty acids, in the hiPSC medium significantly enhances mitochondrial remodeling, the rate of oxygen consumption, as well as the production of ATP. Metabolomic analysis after fatty acid supplementation has demonstrated that fatty acid oxidation increases ATP, which is consistent with the presence of the linkage complex, intercalated discs, t-tubule-like structures, and adult cardiac troponin T isoforms [14]. On the contrary, day-30 CMs, which are maintained by glucose, show an immature ultrastructure and undeveloped bioenergetics, which are affected by poorly developed mitochondria. In hiPSC-derived CMs, the advanced metabolic phenotype that prioritizes fatty acids was achieved, whereby fatty acid-driven-oxidation sustained cardiac bioenergetics and respiratory capacity, contributed to ultra-structural and functional characteristics similar to healthy adult-like CMs [14].

## 3. Different Types of Fatty Acids have Different Roles in the differentiation of Pluripotent Stem Cells into CMs

### 3.1 Effect of Palmitoyl Lipids on the Differentiation of hiPSC into CMs

Induced differentiation and maturation of hiPSC into exogenous, palmitoylated fat-treated CMs results in significant changes in basal and maximal respiration [1, 11]. HiPSC-CMs were sequentially cultured for a week and in maturation medium with fatty acids but no glucose for 3–7 days after differentiation from hiPSCs for 5 days. In a maturation medium containing palmitoyl lipids as the fatty acid, fatty acid oxidation can support ATP production, mimicking the metabolic substrate usage of adult ventricular CMs [4]. The results show that the function of mitochondrial oxidation can be improved and the high capacity of using fatty acids, which is considered as an energy source, can be evidence to infer that metabolic maturation of hiPSC-CMs is enhanced by fatty acid treatment. This contributes to the morphology and structure of cells, the expression of genes and proteins, and the metabolism of cells of hiPSC-CM cultured in the fatty acid-contained medium [1].

### 3.2 The Effect of the Mixture of Linoleic Acid and Oleic Acid on the Differentiation of hiPSC into CMs

The D-glucose-containing medium supplemented with lactate facilitates the purification of hiPSC-CMs. Replacing lactate with linoleic acid-oleic acid-albumin from the beginning of cell culturing is beneficial since free fatty acids are toxic to CM [15, 16] and can be improved by applying the culture medium with Bull Serum Albumin (BSA). This has been shown to bind the free fatty acid, which could be transported to the intracellular space [17, 18]. Fatty acids have similar purification effects to lactate. CMs’ have the ability to use fatty acids and lactate with high efficiency to produce enough association of tennis professionals (ATPS) while non-cardiomyocytes (especially stem cells) could not utilize fatty acid efficiently [19]. Fatty acids also improves the electrophysiological properties of hiPMC-CMs after supplementation of linoleic acid-oleic acid-albumin and 3,3’,5-triiodo-I-thyronine (T3), which is used to potentiate this process [20]. HiPSC-CMs has an enhanced maximal upward velocity of action potentials, action potential amplitude, and repolarization at 50% and 90% of the action potential duration [21]. The treated CMs have greater sensitivity and lower value-added activity to isoproterenol. Expression profiles have shown that various ion channels and myocardial-specific genes are also elevated in CMs, resulting in more mature CMs [19].

### 3.3 Phosphatidylcholine’s Effect on hiPSC-CM Differentiation

Phosphatidylcholine (PC) in fatty acids is important for hiPSCs’ survival. Targeted proteomics has been used to show that fatty acid biosynthesis-related enzymes, including ATP citrate lyase and fatty acid synthase, are expressed in undifferentiated hiPSCs, which are different from those expressed in hiPSC-CMs. Based on past research, in some cell lines, the accumulation of ceramides (Cer), which are regarded as proapoptotic lipids, after fatty acid synthase (FASN) inhibition contributes to cell death [22]. Detailed lipid analysis has shown that inhibition of FASN results in an important reduction in sphingolipids and PC [23, 24]. Furthermore, PC is a major ingredient of lipid bilayers such as cellular membranes. It is produced via de novo FA synthesis and increases during cytokinesis [25]. It is shown in some studies that the effect of FASN inhibition can be attenuated by exogenous PC, which demonstrates that the decrease in PC is of great significance to cell death due to FASN inhibition [23]. However, different from the lipid profiles of hiPSCs, PC was not significantly changed in hiPMC-CMs after treatment with orlistat. This suggests that PC’s reduction in the undifferentiated hiPSCs depends on de novo FA synthesis for proliferation, and demonstrates the important role of PC production via de novo FA synthesis in cytokinesis [25].

### 3.4 Effect of Nitro-Oleic Acid on the Differentiation of hiPSCs into CMs

Nitro-oleic acid is a mediator of pluripotency and has recently been described as an activator of signal transduction and transcriptional activator protein 3 activity [26]. Nitro-oleic acid may also be involved in the regulation of differentiation. Exogenous nitro-oleic acid was added at the beginning of hiPSC culturing. Nitro-oleic acid regulates pluripotency in embryonic stem cells by modulating Stat3 phosphorylation, which induces cardiac-specific gene expression and suppresses cardiac differentiation [26].

### 3.5 Effect of Polyfluoroalkyl Substances on the Differentiation of hiPSCs into CMs

Polyfluoroalkyl substances, including perfluorooctane sulfonate (PFOS) and perfluorooctanoic acid (PFOA), are common persistent contaminants in human blood. At non-cytotoxic concentrations, PFOS and PFOA strongly affect CM differentiation, with perfluoro-1-octane sulfonyl fluoride (PFOSF) being more potent than PFOA. Transcriptional analysis of CM mRNAs has shown that adding exogenous PFOS to the cell culture medium increases the expression of the early cardiac marker islet 1 (ISL1), while decreasing the expression of the CM marker myosin heavy chain 7 (MYH7). This suggests that PFOS, as well as perfluorooctanoic acid, interfere with CM differentiation by disrupting molecular pathways like those induced during embryonic development [27].

### 3.6 Valproic Acid’s Effect on hiPSC Differentiation into CMs

Valproic acid induces global histone H3 acetylation. Core histone modifications induce a significant increase in nucleosome stability and enrichment of sites associated with cytoskeletal organization and cell morphogenesis. Changes in chromatin accessibility are evident at several important genomic loci, including the pluripotency factor *Lefty*, cardiac troponin *Tnnt 2*, and homologous structural domain factor *Hopx*, which play a major role in the duration of CM differentiation [28]. Additionally, valproic acid increases the ability of pluripotent stem cell-derived mesodermal progenitor cells to form myotubes [29]. The therapeutic roles of essential fatty acids and their metabolites in coronary heart disease and hypertension, in addition to their ability to inhibit inflammation, may be related to their capability to proliferate embryonic stem cells and differentiate CMs [30].

## 4. Oxidation of Fatty Acids also Affects the Differentiation and Maturation of hiPSCs into CMs

### 4.1 CMs Metabolic Properties in Adults

The continuous rhythmic contraction of cardiac muscle cells requires a large amount of energy expenditure. Due to insufficient energy reserves, the heart has to constantly produce ATP at a high rate [31]. Under normal conditions, the CM prefers fatty acid oxidation as a source of energy [32]. In a healthy adult heart, mitochondrial oxidative phosphorylation accounts for approximately 95% of ATP production, with fatty acid oxidation accounting for 40%–70% [33, 34, 35]. The fatty acid metabolic profile is unique in embryonic stem cells that drive CMs. The ability of hiPSCs to self-renew is linked to specific metabolic pathways.

### 4.2 Fatty Acid Metabolism is Important in the Differentiation of hiPSCs into CMs

Many studies have shown that anaerobic glycolysis can be used by undifferentiated hiPSCs to satisfy their energy demands even if oxygen availability is sufficient. The hiPSCs’ self-renewal ability appears to be regulated by metabolic pathways that are essential to maintaining this state [36, 37, 38, 39]. ATP is produced by mitochondrial respiration from lipids in the form of fatty acids. Fatty acid and glucose metabolisms, as well as mitochondrial respiration, appear to be critical for embryonic stem cell-derived CMs compared with undifferentiated embryonic stem cells. Therefore, the energy substrate metabolism during cardiac maturation and differentiation is flexible. During the duration of CM maturation and differentiation, the contribution of anaerobic glycolysis to ATP synthesis falls rapidly, while mitochondrial respiration dependent on fatty acids plays an increasingly significant role. The metabolism of induced hiPSC-CMs is more like that of fetal CMs compared with the adult CM phenotype. This suggests that metabolic switches during *in vitro* differentiation are unlikely to have entirely developed to the metabolic state of adult-like CMs. Therefore, it is possible to gain new insights into stem cell differentiation and develop recent strategies for stem cell differentiation using defined medium by identifying cell-specific metabolic pathway components or metabolic pathways [40].

### 4.3 Fatty Acid Oxidation Induces a Mature Metabolic Phenotype

The study of hiPSCs derived from myocardial substrate metabolism, gene expression, and the changes of mitochondrial oxygen consumption by using oleic acid salt and the agonist wY-14643 active peroxidase body multiplication body activated receptor alpha, stimulates the interaction between increasing fatty acid oxidation and mature CMs. CMs derived from hiPSCs show decreased glycolysis and increased fatty acid oxidation. These results demonstrated that the hiPSC-CM profile showed growth and hypertrophy compared with untreated cells, suggesting that fatty acid oxidation induced a more mature metabolic phenotype *in vitro*. In addition, RNA sequencing demonstrated that fatty acid treatment upregulates genes involved in fatty acid b-oxidation and downregulates genes in lipid synthesis and glucose metabolism, specifically, the mRNA level of *CD36*, *CPT-1B*, and *PDK4*, which likely enhance the ability to oxidize fatty acids and make the hiPMC-CM similar to adult CMs. In summary, these studies have provided convincing proof that the metabolic switch from glucose to fatty acids is a driver of hiPSC-CM maturation [41].

### 4.4 Fatty Acid Oxidation Promotes the Development of CMs Characteristics

The utilization of fatty acids as a priority for producing ATP means the advanced metabolic phenotype of developing CMs. In hiPSC-CMs, supplementation with CM and palmitic/oleic acids dramatically enhances mitochondrial remodeling, oxygen consumption rates, and the production of ATP [42]. Metabolomic analysis following fatty acid supplementation has shown elevated levels of ATP promoted by ß-oxidation. In hiPSC-CMs, a higher metabolic phenotype of preferential fatty acid use is achieved, whereby fatty acid-driven ß-oxidation maintains the bioenergetic and respiratory capacity of the heart, contributing to ultrastructural and functional characteristics similar to those of late-developing CMs. Further research on the mitochondrial bioenergetics and ultrastructural adaptations associated with fatty acid metabolism is of great importance in cardiac physiology studies pertaining to late mitochondrial and metabolic adaptations [14].

### 4.5 Fatty Acid Oxidation and the Induction of hiPSCs Differentiation into CMs are Mutually Reinforcing

Fatty acid oxidation is triggered by the peroxisome proliferator-activated receptor α. Peroxisome proliferator-activated receptors (PPAR) agonists increase fatty acid and glucose oxidation as well as cardiac gene expression during cell differentiation, implying a mutually reinforcing relationship between CM metabolism and differentiation such as the *TFPa/HADHA* gene, which is required for fatty acid β-oxidation and cardiolipin re-modeling in human CMs [43, 44].

## 5. The Effect of Excess Fatty Acids on hiPSC Differentiation into CMs 

Moderate amounts of fatty acids promote the differentiation and maturation of hiPSCs into CMs, but excessive amounts of fatty acids can have a negative effect on differentiation. CD36 is a membrane protein that improves the uptake of fatty acids into CMs [45]. Myocardial fatty acid utilization is also governed by the CD36-mediated uptake step [45, 46, 47]. Insulin is triggered via Akt signaling by the translocation of CD36 to the sarcolemma, resulting in an increased rate of cellular fatty acid uptake [48]. Upon the disappearance of such triggers, CD36 is internalized (within minutes) and the fatty acid uptake rate is normalized [49]. Cardiac insulin resistance results in almost total dependence on fatty acids, with little contribution from glucose and other fuel sources [50]. Since fatty acid uptake exceeds metabolic energy requirements, excess fatty acids are stored in triacylglycerols within cells. Through increased levels of lipid metabolites such as diacylglycerols and ceramides, ectopic lipid storage causes insulin signaling inhibition, which can have a negative influence on the differentiation of hiPSCs into CMs [51]. Excess lipid supply can lead to loss of cardiac insulin sensitivity, resulting in loss of CM endothelial acidification and thus impairment of v-ATPase function [52].

## 6. Issues and Prospects

In general, fatty acids affect the differentiation of hiPSC-CMs by influencing organelles, mainly mitochondria, which play a major role in the maturation of CMs [53], cell structure, genetic phenotype, and metabolism. However, there are many different types of fatty acids, and there is not much available research about the influence of other kinds of fatty acids on the differentiation as well as the maturation of hiPSCs into CMs. There is also little information available about the interactions of fatty acids with other classes of substances.

The differentiation of hiPSCs into CMs is a cutting-edge technology that benefits cardiac patients suffering from myocardial infarction and heart failure, leading to opportunities for CM regeneration and repair as well as research challenges. Knowledge of the mechanisms of induced differentiation remains very limited. This is because during the course of hiPSC-CM differentiation, the hiPSC not only differentiates into hiPSC-CMs, but also into many other types of c, such as stem cells, endothelial cells, and fibroblasts. The recent strategy of purification is that hiPSC-CMs are purified at day 20 by culturing them with lactate purification medium for 7 days to eliminate non-CM cells [54]. This method is based on the distinctive biochemical differences between CMs and non-CMs involving lactate and glucose metabolism. While non-CMs rely on glucose as the cell’s main energy source, CMs can produce energy from lactate and fatty acids as well [55, 56]. How can FA improve the efficiency of induction of hiPSCs to hiPSC-CMs and increase enrichment and purification of induced CMs? Furthermore, how can FA control the directional differentiation of hiPSCs, since FA can stimulate an increase in the number of mitochondria and the expression of CMs’ specific genes [57, 58, 59, 60] and simplify their induction pathways, allowing mass production of hiPSCs. These are areas which are worth studying and will contribute to the future treatment of patients with myocardial infarction and heart disease.

## 7. Conclusions

In conclusion, fatty acids are essential for the differentiation and maturation of hiPSC into hiPSC-CM. In general, when hiPSC-CM begins to differentiate into hiPSC-CM and hiPSC-CM differentiation is relatively mature, hiPSC-CM showed a more mature cell structure, improved respiratory metabolism and CM function, more expression of CM specific gene-phenotype, and cell licardiones after culture with a certain amount of specific fatty acids. Their number and function in the body are more complete, and the expression of CM-specific metabolites and CM-specific markers is more varied. The cells show a more mature state, similar to adult CMs.

Recent studies show that different fatty acids may have different effects (Table 1, Ref. [1, 2, 10, 11, 12, 14, 19, 21, 22, 23, 26, 29, 30]). The appropriate amount of exogenous palm grease can make hiPSC-CM differentiated by hiPSC-CM more mature. Phosphatidylcholine is a key metabolite for hiPSC-CM survival, and nitro-oleic acid has a weakening effect on hiPSC-CM differentiation. PFOS and perfluorooctanoic acid interfere with CM differentiation by disrupting molecular pathways evoked during embryonic development. Fatty acid metabolism, as the main metabolic mode of adult CM, can also affect the differentiation and maturation of hiPSC-CM. It can not only induce the more mature metabolic phenotype of hiPSC-CM, and promote the characteristic development of hiPSC-CM, but also promote the differentiation process of hiPSC-CM. At the same time, excess FA often harms the differentiation and maturation of hiPSC-CM. We are not yet able to generate hiPSC-CM consistent with adult CMs. Therefore, we continue to try different kinds of specific fatty acids or mixtures of fatty acids, and other specific external conditions such as physical stretch, electrical stimulation, and a hypoxia environment, to explore the cultivation of hiPSC-CM consistent with adult CMs.

**Table 1.** **Different types of fatty acids have different roles in the differentiation and maturation of pluripotent stem cells into CMs**.

		Effect of fatty acids on the induction of stem cell differentiation into mature CMs	Effect of fatty acids on the induction of stem cell maturation into mature CMs
Different types of fatty acids	Palm fat	Cell expression of genes and proteins, morphology, and structure, and metabolic maturation of hiPSC-CMs cultured in the fatty acid-containing medium [1] and cell elongation increased [10, 12]	Sodium-potassium channels and troponin T exhibit characteristics of mature adult CM with increased numbers [2, 10, 14], oxidative function of mitochondria is developed and maximum respiratory and reserve respiratory capacity and structural and functional characteristics and respiratory capacity are similar to normal CM in adults [1, 11]
	A mixture of linoleic and oleic acids	Action potential amplitude was enhanced, and various ion channels and myocardial-specific genes were also elevated in CMs [19]	The maximum rate of action potential rises, the cells’ mitochondrial function improved and maximum respiratory and reserve respiratory capacity and fatty acid-dependent mitochondrial respiration increases dramatically during maturation [21]
	Phosphatidylcholine	Important for the survival of hiPSCs [22, 23]	Important for the survival of hiPSCs [22, 23]
	Nitro-oleic acid	Induces cardiac-specific gene expression and inhibits cardiac differentiation [26]	Inhibits cardiac maturation
	Polyfluoroethylene substance	Interference with CM differentiation	Inhibits cardiac maturation
	Valproic acid	Increased the ability of pluripotent stem cell-derived mesodermal progenitor cells to form myotubes [29] and promotes embryonic stem cell proliferation and mesodermal differentiation related [30]	The number of mitochondria of hiPSC-CMs increased [10], structural and functional characteristics and respiratory capacity are similar to normal CM in adults and fatty acid-dependent mitochondrial respiration increases dramatically during maturation
